# Antidiabetic pharmacotherapy and anamnestic hypoglycemia in a large cohort of type 2 diabetic patients - an analysis of the DiaRegis registry

**DOI:** 10.1186/1475-2840-10-66

**Published:** 2011-07-14

**Authors:** Diethelm Tschöpe, Peter Bramlage, Christiane Binz, Michael Krekler, Tanja Plate, Evelin Deeg, Anselm K Gitt

**Affiliations:** 1 Herz- und Diabeteszentrum Nordrhein-Westfalen in Bad Oeynhausen, Universitätsklinik der Ruhr Universität, Bochum, Germany; 2 Institut für Kardiovaskuläre Pharmakologie und Epidemiologie, Mahlow, Germany; 3 Bristol Myers Squibb, Medical Department, Munich, Germany; 4 AstraZeneca, Medical Department, Wedel, Germany; 5 Institut für Herzinfarktforschung Ludwigshafen an der Universität Heidelberg, Ludwigshafen, Germany

## Abstract

**Background:**

We aimed to identify predictors of anamnestic hypoglycaemia in type-2 diabetic patients on oral mono- or dual oral combination antidiabetic pharmacotherapy.

**Methods:**

DiaRegis is a prospective registry in type-2 diabetic patients in primary care. Odds ratios (OR) with 95% confidence intervals were determined from univariate logistic regression. Using multivariate logistic regression analysis with stepwise backward selection at an alpha of 0.05 independent predictors of hypoglycaemia were determined.

**Results:**

3,808 patients had data on hypoglycaemia available (median age 65.9 years, 46.6% female). 10.8% had at least one anamnestic hypoglycaemic episode within the previous 12 months. Patients with hypoglycaemia received more sulfonylureas (OR 2.16; 95%CI 1.75-2.67) and less metformin (OR 0.64; 95%CI 0.50-0.82). On top of metformin, patients with thiazolidine (OR 0.50; 95%CI 0.28-0.89) and DPP-4 inhibitor use (OR 0.34; 95%CI 0.16-0.70) had a decreased risk for hypoglycaemia while it was again increased with sulfonylureas (OR 2.08; 95%CI 1.44-2.99). Age < 65 years was an independent predictor of a reduced hypoglycaemia incidence (OR 0.76; 95%CI 0.59-0.96), low Hb_A1c _(OR 1.68; 95%CI 1.31-2.14), stroke/TIA (OR 1.72; 95%CI 1.08-2.72), heart failure (OR 1.77; 95%CI 1.28-2.45), and the use of sulfonylureas (OR 2.58; 95%CI 2.03-3.29) were independent predictors of increased risk.

**Conclusions:**

The results indicate that the risk of hypoglycaemia might be substantially reduced by carefully selecting antidiabetic pharmacotherapy in patients with type-2 diabets in primary care.

## Background

Hypoglycaemia is a serious and potentially life-threatening side effect of antidiabetic drug therapy. The incidence depends on Hb_A1c _targets [1-3] and the specific drug or drug-drug combination prescribed [4]. Patients on insulin are at particularly high risk for hypoglycaemia compared to patients on oral antidiabetic drug therapy. In the UKPDS the incidence of any hypoglycaemic event in insulin-treated newly diagnosed type 2 diabetic patients was 36.5 per 100 patient-years which was at least twice as much as in sulfonylurea-treated patients [5]. The incidence of severe hypoglycaemia was 2.3 per 100 patient-years which was a four- to sixfold increase compared with the sulfonylurea-treated group. This is the more disturbing since sulfonylureas have been shown to confer the highest risk of hypoglycaemia among oral antidiabetic drugs.

Severe hypoglycaemia was considered responsible for excess deaths in the ACCORD trial [6]. Nineteen of the 41 excess deaths from cardiovascular causes in the study were attributed to "unexpected or presumed cardiovascular disease," which may plausibly be related to or may have been precipitated by hypoglycaemia and misclassified as having a cardiovascular cause. Combination therapies, such as a sulfonylurea with insulin, are known to be associated with an increased risk for hypoglycaemia and appear to have been used routinely in this study. This is consistent with a recent analysis of a large cohort study [7], which has shown that mortality is lowest in patients with a Hb_A1c _level of 7.5% and increased below this levels, more so in patients on insulin than in patients on oral antidiabetic therapy. Later analyses of ACCORD however suggested that while symptomatic, severe hypoglycaemia was associated with an increased risk of death, it was not responsible for the difference between intensive and standard therapy [8].

Patients with type-2 diabetes who do not meet treatment targets while receiving oral mono- or dual oral combination therapy are at an increased risk for diabetes and treatment related complications. To identify predictors of incident hypoglycaemia in this patient population we analyzed the dataset of the cross-sectional part of the *Diabetes Treatment Patterns and Goal Achievement in Primary Diabetes Care *(DiaRegis) Registry.

## Methods

DiaRegis is a prospective, observational, German, multicenter registry. The study protocol and baseline characteristics of the patient population have been published [9,10]. This registry is conducted in accordance with Good Epidemiology Practices (GEP), and applicable regulatory requirements. The protocol of this registry was approved by the ethics committee of the Landesärztekammer Thüringen in Jena, Germany on March 4^th ^2009. Patients being enrolled into this registry provided written informed consent. It was registered with the database of the *Verband forschender Arzneimittelhersteller *(VFA).

### Patients

Between June 2009 and March 2010 patients with type-2 diabetes aged ≥ 40 years on oral mono or dual oral combination antidiabetic therapy (no injectables such as insulin and GLP-1 analogs) were included in which the treating physician indicated a change of therapy to be necessary. Patients not under regular supervision of the treating physician for the duration of the study, those with type-1 diabetes, pregnancy, diabetes secondary to malnutrition, infection or surgery, with maturity onset diabetes of the young, known cancer or limited life expectancy, acute emergencies, participation in a clinical trial and patients with further reasons that made it impossible or highly problematic for the patient to participate and come to the follow-up visits were excluded from the registry.

### Documentation

Patient characteristics at baseline were entered via a secure website directly into an electronic database at the *Stiftung Institut für Herzinfarktforschung*, Ludwigshafen, Germany. At this stage they were automatically checked for plausibility and completeness. Data from the patient questionnaire (paper version) which was asked to be completed by the patient during the visit were transferred to the responsible CRO *Winicker Norimed GmbH*, Nürnberg, Germany. The questionnaires were scanned and transferred to the *Stiftung Institut für Herzinfarktforschung *for evaluation.

### Definition of endpoints (hypoglycaemia)

Hypoglycaemia was classified as follows. In case of severe hypoglycaemia patients were seeking medical attention or were admitted to hospital because of hypoglycaemia. In case of moderate hypoglycaemia patient experienced symptoms of hypoglycaemia and required assistance from a second person (e.g. a relative or friend), but no attention of a medical professional was necessary. Mild hypoglycaemia was determined from blood glucose measurements (<2.22 mmol/l; 40 mg/dl in any case; 2.22-2.78 or 50 mg/dl in case of symptoms) and defined as being with or without specific symptoms but manageable without foreign help.

For the present analysis all patients with valid information regarding the presence or absence of hypoglycaemic episodes during the last 12 months prior to enrolment were included. These were obtained on an anamnestic basis and multiple episodes with different severities were possible to document.

### Statistical analysis

The statistical analysis was performed using SAS, version 9.1 (Cary, North Carolina, U.S.A.). The distribution of metric variables is described with medians and quartiles. All descriptive statistics are based on available cases. Comparisons were made with the χ^2 ^or Mann-Whitney-Wilcoxon Test. For patient characteristics unadjusted odds ratios (OR) with 95%-confidence intervals were determined from univariate analyses.

Stepwise multivariable logistic regression analysis was used to estimate adjusted odds ratios (OR) with 95% confidence intervals for the incidence of hypoglycaemia. Variables entered into the multivariate model were identified from univariate analysis and included age, diabetes duration, body mass index (BMI), waist circumference, heart failure, depression, triglycerides, fasting and postprandial glucose as well as Hb_A1c_.

## Results

A total of 3,808 patients were available for the present analysis. The incidence of hypoglycaemia of any type and severity was 10.8% (n = 410). 89.2% (n = 3,398) reported to have had no such episode. 48.8% of patients with anamnestic hypoglycemia reported episodes with no specific symptoms and 67.8% episodes with symptoms that were manageable without help (mild hypoglycaemia). 8.3% had symptoms and required help (moderate hypoglycaemia). 3.1% were seeking attention of a medical professional and 2.9% were admitted to the hospital (severe hypoglycaemia).

### Patient characteristics

Patients with hypoglycaemia were older (p < 0.001), had a longer duration of diabetes (p < 0.01), a lower body mass index (BMI; p < 0.0001) and waist circumference (p < 0.001), and had lower triglyceride levels (p < 0.001). Hb_A1c_, fasting and postprandial glucose levels were lower in patients with hypoglycaemia (p < 0.0001; Table 1).

**Table 1:** Patient characteristics and laboratory values at baseline

	With hypoglycaemia (n = 410) %/median (quartile)	Without hypoglycaemia (n = 3,398) %/median (quartile)	p-value*
Age (years)	68.6 (59.0-74.7)	65.7 (57.5-72.7)	<0.001
Women (%)	48.3	46.4	n.s.
Diabetes duration (years)	6.5 (3.3-10.4)	5.5 (2.8-9.2)	<0.01
BMI (kg/m^2^)	29.0 (26.0-33.0)	30.5 (27.0-35.0)	<0.0001
Waist circumference (cm)	104 (95-115)	107 (98-116)	<0.001
Total cholesterol (mg/dl)	204 (174-231)	205 (177-232)	n.s.
HDL-Cholesterol (mg/dl)	48 (41-59)	47 (40-56)	n.s.
LDL-Cholesterol (mg/dl)	118 (95-145)	120 (98-145)	n.s.
Triglycerides (mg/dl)	158 (116-216)	178 (129-246)	<0.001
Hb_A1c _(mg/dl)	7.2 (6.5-8.0)	7.4 (6.9-8.3)	<0.0001
Fasting plasma glucose (mg/dl)	134 (111-162)	143 (121-173)	<0.0001
Postprandial plasma glucose (mg/dl)	172 (145-207)	186 (157-223)	<0.0001

BMI, body mass index; HDL, high density lipoprotein; LDL, low density lipoprotein; Hb_A1c_, glycosylated haemoglobin A1c; * χ^2 ^or Mann-Whitney-Wilcoxon Test.

The prevalence of co-morbid (mostly) vascular disease was similar between patients with or without hypoglycaemia, except for coronary heart disease (OR 1.44; 95%CI 1.13-1.85), prior stroke/TIA (OR 1.61; 95%CI 1.06-2.44), heart failure (OR 1.99; 95%CI 1.42-2.66), amputation (OR 2.48; 95%CI 1.12-5.50), autonomous neuropathy (OR 1.64; 95%CI 1.00-2.67) which were significantly more frequent in patients with a history of hypoglycaemia (Table 2). Also clinically relevant depression was a frequent observation with 11.7% of patients with a history of hypoglycaemia and 4.4% in patients without (OR 2.87; 95%CI 2.02-4.07).

**Table 2:** Concomitant risk factors and disease

	With hypoglycaemia (n = 398), %	Without hypoglycaemia (n = 3,482), %	OR (95%CI)*
Dyslipidemia (%)	60.0	63.7	0.86 (0.69-1.06)
Hypertension (%)	84.6	84.4	1.02 (0.77-1.35)
Coronary heart disease (%)	23.2	17.3	1.44 (1.13-1.85)
Prior stroke/TIA (%)	6.8	4.4	1.61 (1.06-2.44)
Heart failure (%)	16.6	9.1	1.99 (1.49-2.66)
PAD (%)	7.7	5.8	1.36 (0.92-2.02)
Amputation (%)	2.0	0.8	2.48 (1.12-5.50)
Autonomous neuropathy (%)	5.1	3.2	1.64 (1.00-2.67)
Peripheral neuropathy (%)	15.1	14.2	1.07 (0.80-1.43)
Non-prolif. retinopathy (%)	5.5	3.5	1.58 (0.99-2.52)
Proliferative retinopathy (%)	1.0	0.5	2.22 (0.73-6.71)
Blindness (%)	0	0.1	n.a.
Clinically rel. depression (%)	12.0	4.6	2.84 (2.02-4.01)

TIA, transitory ischemic attack; PAD, peripheral arterial disease; n.a., not applicable; OR, odds ratio; CI, confidence interval; *unadjusted.

### Antidiabetic pharmacotherapy

Of all patients 84.0% received metformin, 28.8% sulfonylureas, 2.7% glucosidase inhibitors, 4.5% glinides, 6.3% thiazolidinediones and 4.9% DPP-4 inhibitors. The use of insulin or GLP-1 analogs was not permitted as to the exclusion criteria. 68.6% received monotherapy and 31.4% dual combination therapy.

**Figure 1:**
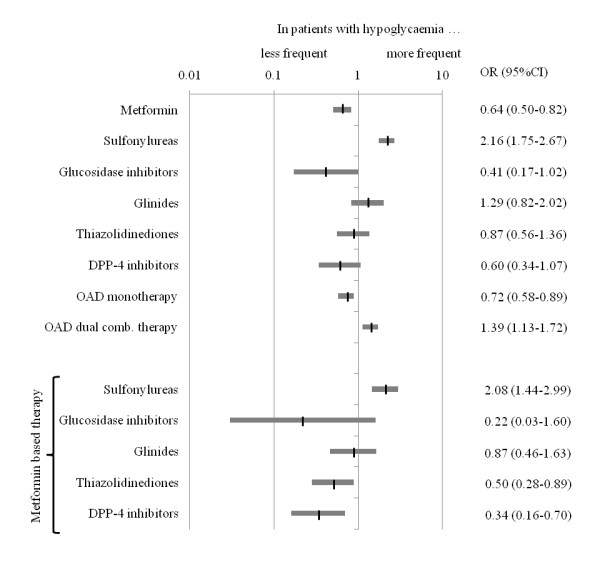
**Antidiabetic pharmacotherapy at baseline overall (upper panel) and metformin based dual combination therapy in patients with and without hypoglycaemia in the past 12 months (lower panel)**. OAD, oral antidiabetic therapy; DPP-4, dipeptidyl peptidase-4; OR, odds ratio; CI, confidence interval.

Patients having experienced hypoglycaemia received monotherapy less (OR 0.72; 95%CI 0.58-0.89) and dual oral combination therapy more frequently (OR 1.39; 95%CI 1.13-1.72) (Figure 1, **upper panel**). With respect to single components metformin was used significantly less (OR 0.64; 95%CI 0.50-0.82) in patients with hypoglycaemia while the use of sulfonylureas was increased (OR 2.16; 95%CI 1.75-2.67). Further there was a strong trend for a reduced hypoglycaemia incidence with glucosidase inhibitors (OR 0.41; 95%CI 0.17-1.02), which was non-significant (low patient numbers).

Figure 1, **lower panel **illustrates that patients reporting hypoglycaemia had frequently received a combination of sulfonylureas with metformin (OR 2.08; 95%CI 1.44-2.99). On the other hand patients receiving DPP-4 inhibitors (OR 0.34; 95%CI 0.16-0.70), thiazolidinediones (OR 0.50; 95%CI 0.28-0.89) and as a trend glucosidase inhibitors (OR 0.22; 95%CI 0.03-1.60) on top of metformin had a decreased risk for hypoglycaemia in univariate analyses.

### Treatment decisions in patients with hypoglycaemia

In 74.4% of patients that had experienced hypoglycaemia the therapy was changed because of suboptimal blood glucose adjustments (OR 0.38; 95%CI 0.30-0.48 vs. no hypoglycaemia), 30.7% because of hypoglycaemia (OR 187.8; 95%CI 90.99-387.8), 15.4% because of weight gain (no difference), 10.5% due to unexpected adverse events (OR 2.00; 95%CI 1.41-2.83) and a further 11.1% for a variety of other reasons (no difference).

**Figure 2:**
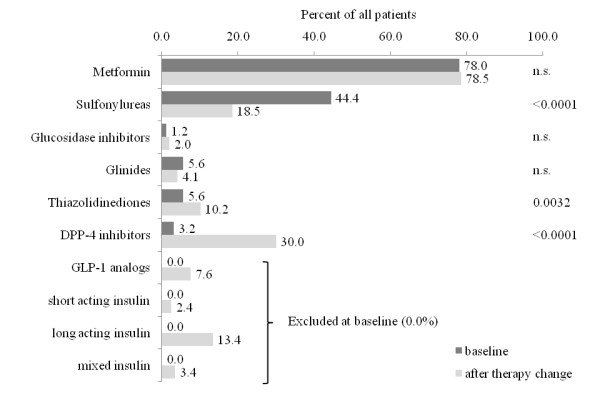
**Antidiabetic pharmacotherapy in patients with hypoglycemia within the last 12 months prior to enrolment and after therapy change**. DPP-4, dipeptidyl peptidase-4; GLP-1, glucagon-like peptide-1; n.s., not significant; p-values calculated by the McNemar's Test.

Compared to baseline the use of sulfonylureas in patients with hypoglycaemia was strongly reduced (-25.9%), while in a significant proportion of patients DPP-4 inhibitors (+26.8%), GLP-1 analogs (+7.6%) and insulins (+26.6%) were introduced (Figure 2). Surprisingly there was a significantly higher degree of insulin use in patients with anamnestic hypoglycaemia (OR 1.88; 95%CI 1.49-2.39), while less oral antidiabetic drugs were prescribed in these patients (mean 1.5 ± 0.7 vs. 1.8 ± 0.7; p < 0.0001).

### Multivariable predictors of hypoglycaemia

Using logistic regression analysis we determined that an age < 65 years (but not gender) was associated with a reduced hypoglycaemia incidence (OR 0.76; 95%CI 0.59-0.96). On the contrary anamnestic a low Hb_A1c _(OR 1.68; 95%CI 1.31-2.14), stroke/TIA (OR 1.72; 95%CI 1.08-2.72), heart failure (OR 1.77; 95%CI 1.28-2.45), and the use of sulfonylureas (OR 2.58; 95%CI 2.03-3.29) were associated with an increased risk for hypoglycaemia.

## Discussion

The treatment algorithm of the German Diabetes Society (Deutsche Diabetes Gesellschaft, DDG) is based on the achievement of Hb_A1c _target levels [11]. When metformin monotherapy is not sufficient because Hb_A1c _remains ≥ 6.5% but is < 7.5% after 3-6 month, a number of different oral antidiabetic drugs are recommended to be added. Insulin is recommended if Hb_A1c _is still ≥ 6.5% after further 3-6 month or if Hb_A1c _is ≥ 7.5% after the initial metformin monotherapy phase. This recommendation is somewhat in contrast with the recommendations of the American Diabetes Association (ADA) which recommends to escalate metformin monotherapy if Hb_A1c _is 7% or higher and recommends to use sulfonylureas (other than glibenclamide or chlorpropamide) or a basal insulin because they are "well-validated core therapies" [12]. As to these guidelines the use of further oral antidiabetic drugs (pioglitazone, exenatide) may be considered when hypoglycaemia is particularly undesirable (e.g. in patients who have hazardous jobs). Their future use is however questionable because of their recent withdrawal in parts of Europe [13]. The remaining drug classes (glinides, glucosidase inhibitors, and DPP-4 inhibitors) get only cursory mention in the ADA guidelines. Finally the EASD guidance is less specific in guiding escalation therapy, but states that metformin usually is the treatment option of choice for both mono- and combination therapy, including insulin [14]. The choice of drugs is based on the glucometabolic situation and co-morbid disease conditions.

### Pharmacotherapy and the risk of hypoglycaemia

The ADA guidelines emphasize the prevention of hypoglycaemia to be critical to the treatment strategy in type 2 diabetes [15]. A drug's rate of hypoglycaemia is therefore to be considered when selecting a drug for treatment. Within this context not only complications of severe hypoglycaemia such as coma, cardiac arrhythmias, or myocardial ischemia deserve mentioning but also the symptoms and long term consequences of mild hypoglycaemia [16]. In placebo controlled studies, patients receiving sulfonylureas or glinides experienced increased rates of hypoglycaemia (RR range, 4.57-7.50) [4]. This was also confirmed in a recent meta-analysis, which reported the incidence of overall hypoglycaemia to be increased when glinides were added to a maximum tolerated dose of metformin (RR 7.92 (95%CI 1.45-43.21), while the risk with sulfonylureas was also elevated but this was not significantly significant (RR 2.63; 95%CI 0.76-9.13) [4]. This increased risk is likely because of an increase in insulin release, which may occur independent of the presence of a glucose load [17]. On the other hand there was a trend for a reduced incidence of hypoglycaemia in the aforementioned meta-analysis, the reduction not reaching significance with α-glucosidase inhibitors (RR 0.60; 95%CI 0.08-4.56), and DPP-4 inhibitors (RR 0.67; 95%CI 0.30-1.50).

The present analysis of the association between the incidence of hypoglycaemia and different drug treatment options confirms these prior findings and documents that the differences observed in clinical trials are highly relevant for clinical practice. Use of sulfonylureas was associated with an increased hypoglycaemia incidence in mono- (OR 1.98; 95% 1.45-2.75) as well as in combination therapy with metformin (OR 2.08; 95% 1.44-2.99), which was even stronger after multiple adjustments for significant differences in patient characteristics at baseline (2.58; 95%CI 2.03-3.29). At a mean overall hypoglycaemia rate of 10.8% and a strongly increased risk with sulfonylureas it appears surprising that 18.5% of patients still receive this drug class after therapy change. While the ADA recommends sulfonylureas as a preferred escalation of metformin monotherapy, the likely reason for its frequent use is not their mentioning in the DDG guidelines but their preference in the German disease management program diabetes, which calls for the use of sulfonylureas. Two limitations of the present dataset with respect to this important question deserve mentioning. First, we did only document drug classes used but not single drugs. This would have been important in view of the perceived differential risk which appears to be higher with glibenclamide or chlorpropamide, but lower with other second-generation sulfonylureas (gliclazide, glimepiride, glipizide etc.) [18,19]. Second no information on the dose of drugs was obtained, which compromises to differentiate whether low doses of sulfonylureas would have been more favourable.

On the other hand we found that metformin use was associated with a lower incidence of hypoglycaemia (OR 0.64; 95%CI 0.50-0.82), the use of glucosidase inhibitors showing a trend for a reduced incidence (OR 0.41; 95%CI 0.17-1.02). DDP-4 inhibitors on top of metformin were associated with a reduced incidence of hypoglycaemia (OR 0.34; 95%CI 0.16-0.70); as were thiazolidinedones (OR 0.50; 95%CI 0.28-0.89). Because of low patients numbers (1.2% of patients) the strong trend for glucosidase inhibitors (OR 0.22; 95%CI 0.03-1.60) was non-significant. This finding is of importance since metformin and DPP-4 inhibitors are related in that metformin has an inhibiting effect on DPP-4 [20]. The benefits of metformin are also supported by a more recent Cochrane analysis [21] and its role as a first line treatment in patients with diabetes is undisputed [11,12] which is underlined by 84.0% of patients receiving metformin in DiaRegis. 4.9% of patients received DPP-4 inhibitors in the present analysis, with the majority in combination with metformin. DPP-4 inhibitors are only effective under conditions of hyperglycaemia and disappear when blood glucose values fall below the normal range. Therefore they have no intrinsic risk of hypoglycaemia. Consequently a meta-analysis of 29 clinical studies, severe hypoglycaemia (defined as hypoglycaemia requiring external assistance) was reported for only 2 patients [22]. This is reassuring, given that in contrast to sulfonylureas DPP-IV inhibitors have been available for about 2 years now and are thus considered to be relatively new. Likewise reduced rates of hypoglycaemia have been reported of glucosidase inhibitors [4]. These reports are however frequently only reporting a trend because patients numbers have been low in the considered trials. The risk of hypoglycaemia is however increased in combination with sulfonylureas (DiaRegis OR 3.39; 95%CI 1.01-11.40), and this is attributable to the effect of sulfonylureas [11].

### Adjustment of pharmacotherapy in patients with hypoglycaemia

The majority of patients enrolled into DiaRegis were to be changed with respect to drug therapy at baseline because of insufficient diabetes control. In patients with anamnestic hypoglycaemia within the last 12 months prior to enrolment, in 30.7% therapy was altered because of hypoglycaemia, the majority because of insufficient blood glucose control (multiple answers possible). While the use of sulfonylureas was strongly reduced after change (18.5 vs. 44.4%), there was a strong preference to introduce DPP-4 inhibitors (+26.8%), GLP-1 analogs (+7.6%) or any insulin (+26.6%) (Figure 2). The reduced prescription of sulfonylureas and the increased use of DPP-4 inhibitors is reasonable given the rationale outlined above. The frequent addition of insulin (most likely due its potent lowering of blood glucose) to the oral antidiabetic drug therapy regimen is however surprising when only considering the risk of hypoglycaemia. For these a higher incidence of hypoglycaemia has been reported in comparison to oral antidiabetics [23]. However, given that in 74.4% of patients, blood glucose adjustment was the main reason for changing therapy, insulin use might be reasonable and is enforced by the guidelines. In the DDG guidance insulin is added if Hb_A1c _is ≥ 6.5% after 3-6 month of mono or multiple oral antidiabetic drug therapy or if Hb_A1c _is ≥ 7.5% after metformin monotherapy [11]. The ADA recommends using insulin (or sulfonylureas) directly if Hb_A1c _is not reduced below 7.0% in patients on metformin monotherapy [12].

## Conclusions

DiaRegis illustrates that there is considerable proportion of patients, experiencing episodes of hypoglycaemia during antidiabetic treatment on mono- or dual oral combination therapy. The incidence is strongly associated to higher age (≥ 65 years), a low Hb_A1c _and heart failure, prior stroke/TIA, but the strongest independent predictor is the use of sulfonylureas. The results indicate that the risk of hypoglycaemia might be substantially reduced by properly selecting antidiabetic pharmacotherapy in a primary care cohort of type-2 diabetic patients.

## Competing interests

Diethelm Tschöpe, Peter Bramlage and Anselm K. Gitt have received research support and honoraria for lectures from Bristol-Myers Squibb and AstraZeneca, the sponsors of the present registry. Christiane Binz, Michael Krekler and Tanja Plate are employees of the sponsors. Evelin Deeg has no potential conflict of interest to disclose.

## Authors' contributions

PB, AKG, DT, CB, MK and TP were involved in the conception and design of the study. ED is responsible for the analysis of data. DT and PB have drafted the manuscript and all other authors have been revising the article for important intellectual content. All authors have finally approved the version to be published.
